# Evaluation and diagnostic potential of circulating extracellular vesicle-associated microRNAs in adrenocortical tumors

**DOI:** 10.1038/s41598-017-05777-0

**Published:** 2017-07-14

**Authors:** Pál Perge, Henriett Butz, Raffaele Pezzani, Irina Bancos, Zoltán Nagy, Krisztina Pálóczi, Gábor Nyírő, Ábel Decmann, Erna Pap, Michaela Luconi, Massimo Mannelli, Edit I. Buzás, Miklós Tóth, Marco Boscaro, Attila Patócs, Peter Igaz

**Affiliations:** 1 2nd Department of Medicine, Semmelweis University, 1088 Budapest, Szentkirályi str. 46., Hungary; 2 Molecular Medicine Research Group, Hungarian Academy of Sciences and Semmelweis University, 1088 Budapest, Szentkirályi str. 46., Hungary; 3 Endocrinology Unit, Department of Medicine, University of Padua, Via Ospedale, 105, 35128 Padova, Italy; 4 Division of Endocrinology, Diabetes, Metabolism and Nutrition, Department of Internal Medicine, Mayo Clinic, 200 First Street SW, Rochester, MN 55905 USA; 5 Department of Genetics, Cell- and Immunobiology, Semmelweis University, 1089 Budapest, Nagyvárad tér 4., Hungary; 6 Department of Experimental and Clinical Biomedical Sciences, Endocrinology Unit, University of Florence, Viale Pieraccini 6, 50139 Florence, Italy; 7 “Lendület-2013” Research Group, Hungarian Academy of Sciences and Semmelweis University, 1088 Budapest, Szentkirályi str. 46., Hungary

## Abstract

There is no available blood marker for the preoperative diagnosis of adrenocortical malignancy. The objective of this study was to investigate the expression of extracellular vesicle-associated microRNAs and their diagnostic potential in plasma samples of patients suffering from adrenocortical tumors. Extracellular vesicles were isolated either by using Total Exosome Isolation Kit or by differential centrifugation/ultracentrifugation. Preoperative plasma extracellular vesicle samples of 6 adrenocortical adenomas (ACA) and 6 histologically verified adrenocortical cancer (ACC) were first screened by Taqman Human Microarray A-cards. Based on the results of screening, two miRNAs were selected and validated by targeted quantitative real-time PCR. The validation cohort included 18 ACAs and 16 ACCs. Beside RNA analysis, extracellular vesicle preparations were also assessed by transmission electron microscopy, flow cytometry and dynamic light scattering. Significant overexpression of *hsa-miR-101* and *hsa-miR-483-5p* in ACC relative to ACA samples has been validated. Receiver operator characteristics of data revealed dCT_*hsa-miR-483-5p*_ normalized to *cel-miR-39* to have the highest diagnostic accuracy (area under curve 0.965), the sensitivity and the specifity were 87.5 and 94.44, respectively. Extracellular vesicle-associated *hsa-miR-483-5p* thus appears to be a promising minimally invasive biomarker in the preoperative diagnosis of ACC but needs further validation in larger cohorts of patients.

## Introduction

Adrenocortical tumors are common in humans, and their prevalence rises with age^1^. These are mainly represented by benign adrenocortical adenomas (ACA). The majority of ACA is hormonally inactive and thus clinically indolent, but hormone-producing ACAs secreting cortisol or aldosterone are associated with serious morbidity and increased mortality^1^. In contrast, adrenocortical carcinoma (ACC) is a rare, but aggressive neoplasm. The incidence of ACC is about 0.5-2 cases per million people per year^2, 3^. The prognosis of ACC is poor as the estimated 5-year survival ranges from 15 to 30% in advanced stages^4^. There is no reliable preoperative marker for distinguishing ACA from ACC at present. Imaging modalities have considerable limitations^5^ and biopsy is not recommended due to difficulties of histological analysis and fear for tumor spread^4^. Urinary steroid metabolomics is a promising approach for preoperative diagnosis of malignancy, but is not widely available and requires a 24 h urine collection^6^.

Recent studies have reported significantly altered expression of both tissue and circulating microRNAs (miRNAs) in ACC versus ACA^7–12^. MiRNAs are short (19–24 nucletide long) non-protein coding RNA molecules involved in the regulation of gene expression primarily as endogenous mediators of RNA interference^13^. Beside tissue miRNAs, novel studies proved the stable existence of miRNAs in different body fluids^14^, as well. These extracellular miRNAs could serve as minimally invasive biomarkers of malignancy and prognosis in different tumors^15, 16^. In our previous study, we found that circulating miRNAs isolated from whole plasma could serve as potential biomarkers for adrenocortical carcinoma^10^. However, the diagnostic sensitivity and specificity were not high enough for clinical applicability^10^. In the blood plasma, miRNAs are found both in extracellular vesicles (EV) (such as microvesicles, exosomes, apoptotic bodies) and in macromolecular complexes with lipoproteins^17^ or the Argonaute 2 protein^18^. The mechanism for cellular miRNA release is only partially understood, but the active secretion of miRNA in extracellular vesicles appears to be a regulated process^19^, and thus, might be directly linked to disease pathogenesis. Exosomes represent a major class of extracellular vesicles that are formed via the endo-lysosomal pathway and are 40–100 nm in diameter^19^. Exosomal miRNAs might even be implicated in cell-to-cell communication^20^. Whereas circulating miRNAs isolated from whole plasma include miRNAs released due to tissue damage or necrosis^21^, we hypothesize that miRNAs secreted actively in EVs could be more specific as minimally invasive biomarkers. The objective of this study was to investigate the expresssion of EV-associated miRNAs and their diagnostic potential in patients with adrenocortical tumors. To the best of our knowledge, this is the first study to investigate the biomarker potential of extracellular vesicle-associated microRNAs in adrenocortical tumors.

## Results

### Confirming the nature of isolated extracellular vesicles

We have used both transmission electron microscopy and flow cytometry to confirm the presence of EVs in our samples to fulfill the minimal experimental requirements for extracellular vesicles^22^.

**Figure 1:**
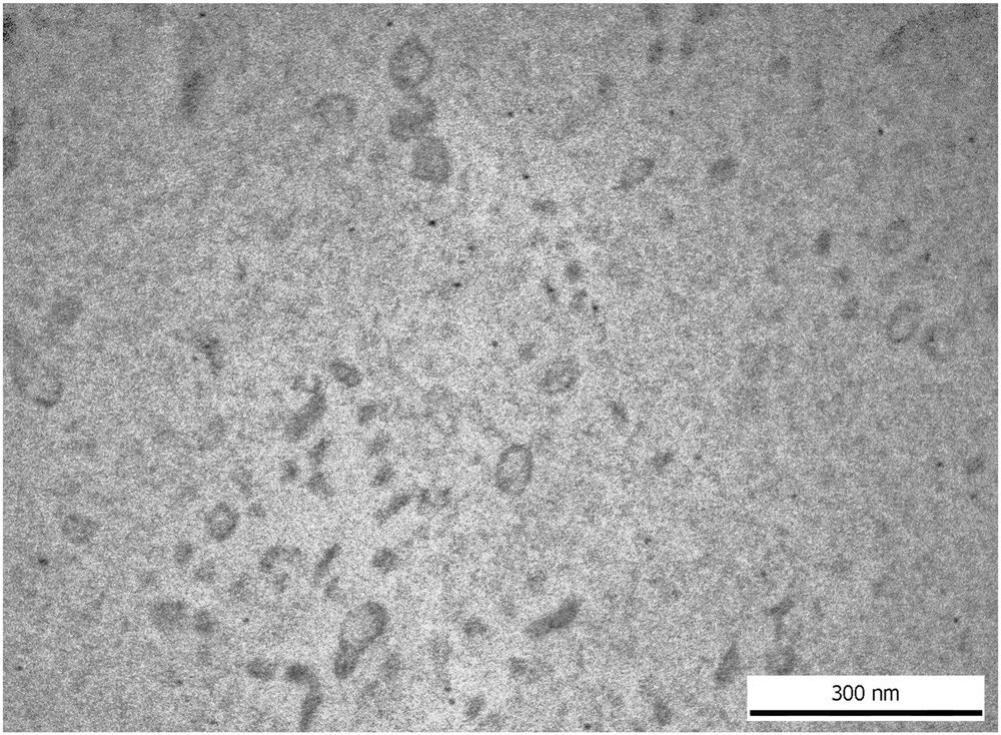
Transmission electron microscopic image of human blood plasma EVs isolated by ultracentrifugation, washed once and submitted to RNAse digestion.

Transmission electron microscopy microphotographs taken from EVs confirmed that the EV size and morphology corresponded to those described for exosomes (Fig. 1).

**Figure 2:**
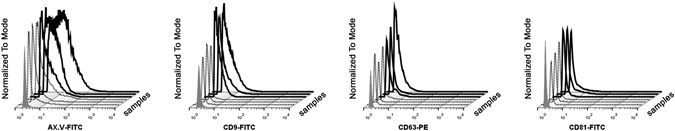
Flow cytometry detection of surface markers of human platelet free blood plasma extracellular vesicles (EVs) conjugated onto latex beads. EVs were isolated either by Total Exosome isolation kit (Life Technologies, by Thermo Fisher Scientific, Waltham, MA, USA) (empty histograms with dotted lines) or ultracentrifugation at 100,000 g (empty histograms with continuous lines) from three different human plasma samples, respectively. Antibody binding to BSA-coated latex beads is shown in gray histograms.

Flow cytometric analysis of EVs isolated by ultracentrifugation confirmed the presence of CD9, CD63, CD81 membrane proteins and annexin V cytosolic protein (Fig. 2). In the vesicle preparations isolated by Total Exosome Isolation (from plasma) Kit, we could identify CD9, CD81 and annexin V (Fig. 2). The percentage of positive beads is presented in Supplementary Information Table S1.

### Size distribution of the isolated extracellular vesicles

**Figure 3:**
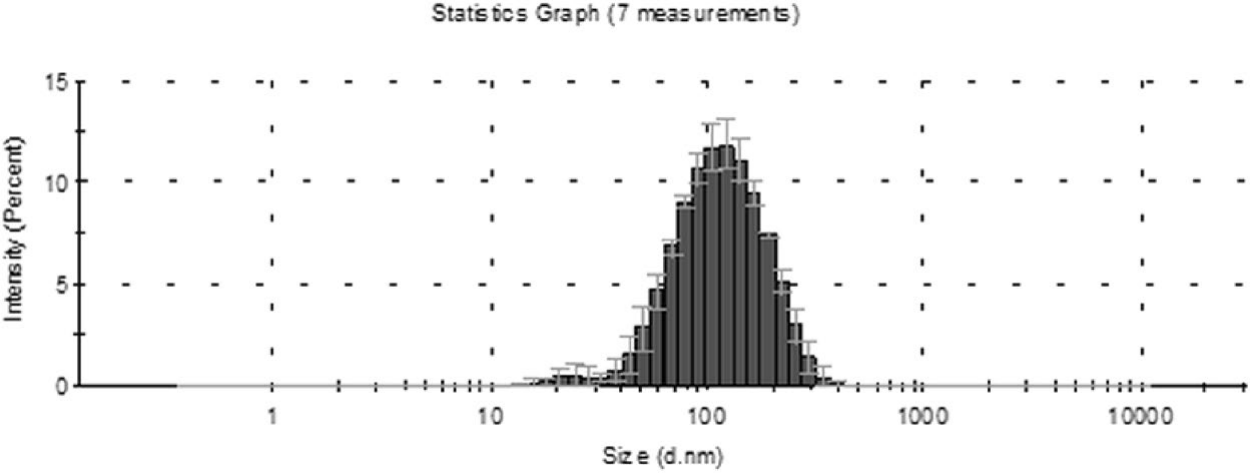
Representative size distribution of EVs isolated by Total Exosome Isolation Kit (Life Technologies, by Thermo Fisher Scientific) in sample 4 (Table 1). The y axis shows the intensity percentage of the vesicles, and the x axis shows the vesicle diameter (nm). The sizes of the EVs were approximately in the range of 16–255 nm (diameter [mean ± SD], 90.08 ± 2.45).

We have used the dynamic light scattering technique to measure the size distribution of extracellular vesicles. The vesicles were isolated from 4 samples by Total Exosome Isolation (from plasma) Kit. The Z-average value of the 4 samples is 80.83 ± 19.07 nm in PBS at 25 °C. The detailed results of the dynamic light scattering are summarized in Table 1. The representative size distribution of EVs in sample 4 is presented in Fig. 3.

**Table 1:** Results of dynamic light scattering.

Sample Name	PDI	Standard deviation of PDI	Z-average (diameter nm)	Standard deviation of Z-average	Intensity mean (diameter nm)	Standard deviation of Intensity mean)
Sample 1	0.21	0.01	97	1.06	122.9	1.77
Sample 2	0.2	0.01	98.6	0.76	124.1	2.77
Sample 3	0.25	0.01	62.8	0.77	85.9	1.85
Sample 4	0.26	0.01	65	0.3	90.8	2.45

### Screening by miRNA expression profiling

To identify differences in circulating EV-associated miRNA expression profiles of ACA and ACC patients, 12 samples have been subjected to TaqMan Array Human MicroRNA A card analysis (6 ACA and 6 ACC plasma samples). Altogether 377 human miRNA have been evaluated. By applying straightforward statistics (described in the Methods section) and Benjamini-Hochberg false discovery rate (FDR) correction, we have found no significant differences in miRNA expression between the two groups. However, we used TLDA data as an initial screening on a limited number of samples and found that 32 miRNAs are expressed in all samples of both ACA and ACC groups. We identified no miRNA expressed only in ACA or ACC groups, either. By using Fisher’s exact test, we evaluated miRNAs which were not expressed in all samples of a group and we found 2 miRNAs showing a tendency being different between ACA and ACC samples: *hsa-miR-101* and *hsa-miR-483–5p*. These have been selected for further validation. *Hsa-miR-101* was expressed in 1 of 6 ACA and 5 of 6 ACC samples, whereas *hsa-miR-483-5p* in 2 of 6 ACA and in all ACC samples. Raw profiling data are presented in Supplementary Dataset file 1.

### miRNA Validation by RT-qPCR

**Figure 4:**
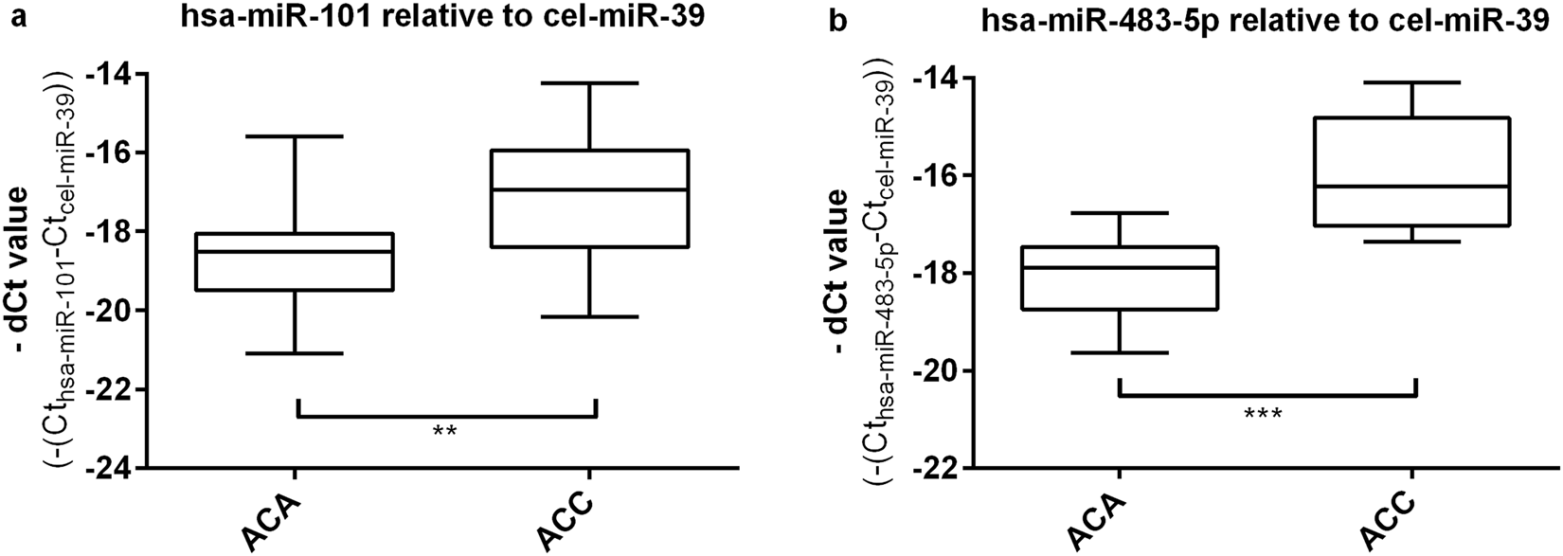
Results of RT-qPCR validation of *hsa-miR-101* (**a**) and *hsa-miR-483-5p* (**b**) normalized to the housekeeping *cel-miR-39*, (mean ± SD, **p < 0.01, ***p < 0.001; unpaired t-test; n = 18 ACA, n = 16 ACC.

The validation cohort included 18 ACAs and 16 ACCs. The widely used synthetic spike-in control RNA *cel-miR-39 was applied* as reference gene^16, 23–25^. We have found significant overexpression of *hsa-miR-101* (Fig. 4a) and *hsa-miR-483-5p* (Fig. 4b) and in ACC relative to ACA plasma EV samples (p < 0.0001 and p < 0.0052, respectively). Raw and normalized qRT-PCR data are presented in Supplementary Dataset files 2 and 3.

### Diagnostic Performance of miRNA

**Figure 5:**
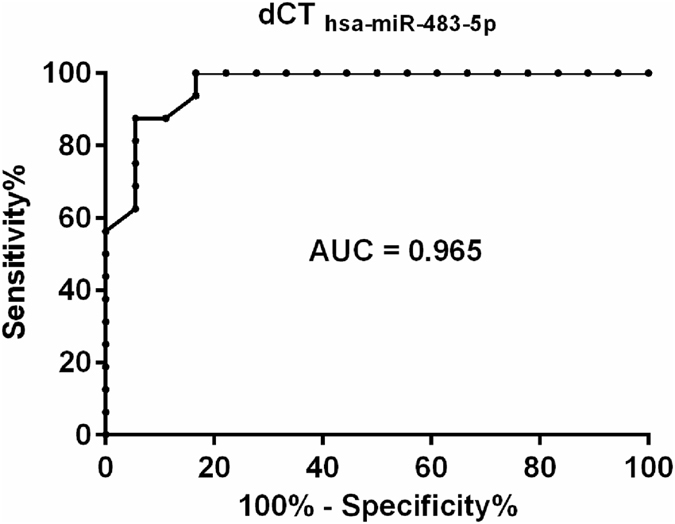
Receiver operating characteristics (ROC) curve analysis for the dCT_*hsa-miR-4835-p*_ exosomes miRNA signature relative to housekeeping *cel-miR-39* based on the results obtained from RT-qPCR analysis of ACA (n = 18) and ACC (n = 16) plasma exosomes samples.

We have evaluated the potential application of EV-associated miRNAs as minimally invasive biomarker candidates of adrenal malignancy by ROC analysis. Both, *hsa-miR-101* and *hsa-miR-483-5*p, relative to spike-in control *cel-miR-39* have been analyzed by ROC analysis. The dCT_*hsa-miR-483-5p*_ relative to *cel-miR-39* showed the highest area under curve (AUC) value (0.965). By setting the cutoff point to 17.25, the sensitivity and the specificity of the test to discriminate ACA and ACC were 87.5 and 94.44, respectively (Fig. 5). ROC data of *hsa-miR-101* have not yielded promising sensitivity and specificity values. By setting the cutoff point to 17.82 the AUC value was 0.766, with 68.75 sensitivity and 83.33 specificity.

### miRNA expression analysis in samples prepared by ultracentrifugation

**Figure 6:**
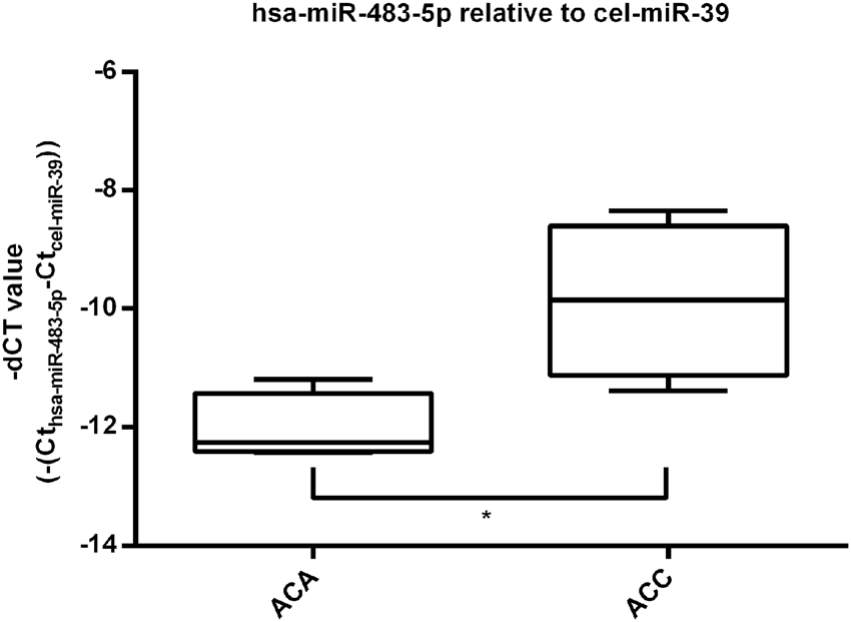
Results of ultracentrifugation with RT-qPCR of *hsa-miR-483-5p* normalized to the spike-in control *cel-miR-39*. Results are represented by – dCT (cycle threshold) (mean ± s.d., *p < 0.05; unpaired t-test; n = 4 ACA, n = 4 ACC).

We have evaluated the expression of *hsa-miR-483-5p* in vesicles isolated by ultracentrifugation, as well. The ultracentrifugation protocol has been performed on 4 ACA and 4 ACC patients. For data normalisation we used *cel-miR-39* as reference gene. We have found significanty higher expression of *hsa-miR-483-5p* in exosomes in ACC relative to ACA (p = 0.0221, Fig. 6).

## Discussion

Recent studies indicated that circulating microRNAs could serve as minimally invasive biomarkers of malignancy in different tumors^26^. In our previous study on unfractionated plasma samples, we have found significant overexpression of 5 circulating miRNAs (*hsa-miR-483-5p*, *hsa-miR-100*, *hsa-miR-181b*, *hsa-miR-184*, *hsa-miR-210*) in ACC relative to ACA^10^. Two further studies on circulating microRNAs in unfractionated serum samples from adrenocortical tumor patients have been reported to date^8, 9^. Overexpressed circulating *hsa-miR-483-5p* in ACC has been confirmed in both, along with overexpressed *hsa-miR-34a* ^9^ and underexpressed *hsa-miR-195* ^8^. The sensitivity and specificity values for these circulating microRNAs cannot be regarded as high enough for clinical introduction at present. The best results reported by Chabre *et al*. for circulating *hsa-miR-483-5p* could only be used to differentiate aggressive and non-aggressive ACC^8^.

Multiple studies demonstrated that miRNA content in actively secreted EVs was quite different from that of donor cells^20, 27, 28^. Therefore, it can be hypothesized that, EVs-associated miRNAs might prove to be more sensitive and specific biomarkers for pathological conditions than EV-free miRNAs or miRNAs from unfractionated body fluid samples.

We aimed to investigate the the expression of EV-associated miRNAs and their potential diagnostic applicability in patients suffering from adrenocortical tumors. To the best of our knowledge, our study is the first of this kind in these tumors.

The overexpression of *hsa-miR-483-5p* and *hsa-miR-101* in ACC has been confirmed in the validation cohort. Showing the highest AUC, specificity and sensitivity values, exosomal *hsa-miR-483-5p* could be a potential biomarker of adrenal malignancy using *cel-miR-39* as reference gene. The significant overexpression of *hsa-miR-483-5p* relative to *cel-miR-39* in ACC has been also confirmed by testing EVs isolated by differential ultracentrifugation, confirming the EV-association of this miRNA.

There is no broadly accepted valid reference gene for raw data normalization for circulating miRNA^29^. The small nuclear RNA (snRNA) U6, *hsa-miR-16* and the synthetic, exogenous spike-in control *cel-miR-39* are the most frequently applied reference genes^29^ in different studies. However, snRNA U6 has a significant interindividual variability^30^. In our previous study on plasma samples, we have found that *hsa-miR-16* and *cel-miR-39* were applicable for normalization^10^. In this study, we have also used *cel-miR-39* as reference both for samples isolated by the kit and ultracentrifugation^16, 23–25^. As *cel-miR-39* is not included in TLDA cards, it could not be assessed as reference in the miRNA screening. *Cel-miR-39* can not be considered as a valid biological control, but definitely indicates the efficacy of miRNA isolation^10^.

In order to confirm the results obtained by using Total Exosome Isolation Kit and to fulfill the minimal experimental requirements for EVs, we have isolated EVs also by differential centrifugation/ultracentrifugation, and carried out EV analysis by using TEM and flow cytometry, as well^22^. Ultracentrifugation is considered to be gold standard of EV isolation to date^31^. TEM enabled to evaluate both the size and the morphology of EVs, simultaneously^32^. Using flow cytometry we detected the presence of annexin V, CD9 and CD81 on EVs isolated both by ultracentrifugation and the kit used. Annexin V and the tetraspanins CD9, CD63 and CD81 are considered to be characteristic for EVs^33^. We can thus conclude that the preparations that we isolated both by the kit used and by differential centrifugation/ultracentrifugation were enriched in EVs. From among the different types of EVs, exosomes are the ones that are known to be characterized by the presence of CD63, CD9 and CD81 in their membranes. Thus, we may conclude that our preparations were enriched in exosomes. The size distributon analysis of extracellular vesicles also supports the exosomal enrichment of the preparations. However, given that at present there are no universal markers to distinguish microvesicles form exosomes, in our conclusions we chose to refer to EVs rather than exosomes or microvesicles. It must be noted, however, that the expression of CD9 and CD63 was higher in the EV samples isolated by ultracentrifugation than by the kit used (CD63 was not detected in EV samples isolated by the kit). The plasma sample volumes needed for ultracentrifugation are much higher (3 mL) than the volumes needed for isolation by the kit (200 µl), thus the yield is also higher by ultracentrifugation. However, the ultracentrifugation-based protocol is very laborious and time-consuming, and not easily applicable for clinical purposes. Our aim has been to use a methodology that could be easily used in the clinical setting. Given the parallel results of miRNA expression used both protocols, we reckon that the isolation kit can be reliably used, and could be used in the clinical setting to analyze larger sets of samples.

Several studies highlight the relevance of these miRNAs in different physiological and pathological conditions. Among these, the overexpression of *hsa-miR-483-5p* has been described both in tissue and in blood of patients suffering from ACC^7–9, 34, 35^. In a previous study, we demonstrated that the expression of circulating *hsa-miR-483-5p* was not influenced by treatments with dexamethasone or adrenocorticotropin confirming that it could be potentially used in the preoperative diagnostic of ACC^36^. Furthermore, circulating *hsa-miR-483-5p* could represent not only a diagnostic marker, but a prognostic marker, too, since its expression predicts poor prognosis and cancer reccurence^8^.

To the best of our knowledge, the overexpression of *hsa-miR-101* in ACC has not been reported so far. There are data on the overexpession of *hsa-miR-101* in malignant pheochromocytoma compared to benign tumors^37^. Among others, *miR-101* may have tumor suppressor role e.g. in stomach^38^, colorectal^39^, prostate^40^, tumors that is not suprising for a miRNA considering their tissue specific action. The same miRNA can be oncogenic or tumor suppressor depending on the cellular context^41^.

In our previous study using total plasma as the source of miRNAs, altogether 5 miRNAs proved to be significantly overexpressed in ACC samples^10^. Since only selected miRNAs based on previous studies were included without profiling, the number of significantly differentially expressed miRNA in total plasma might be even higher. In the present study using EV-associated miRNAs, only two could be validated suggesting a possibly more specific, adrenocortical origin of these miRNAs underlining the higher potential of EV-associated miRNAs than those isolated from whole plasma.

In conclusion, our results show that EV-associated miRNAs might be promising candidates to be minimally invasive biomarkers of adrenocortical cancer. We have used both high-throughput analysis for screening and targeted RT-qPCR for validation to investigate the expression of miRNAs, which seems to be the most appropriate method for blood-borne miRNA analysis^42^. However, the limited sizes of patient cohorts, the lack of standardization of EV isolation, miRNA extraction methods and no generally accepted RNA control for normalization makes it difficult to generalize the results of such studies. In this study, we have found high enough sensitivity and specificity for potential clinical application, however, further studies on larger numbers of patients are needed to prove this assumption.

## Materials and Methods

### Patients’ samples

Altogether 46 preoperative plasma samples have been collected from patients with ACA (n = 24) and ACC (n = 22) at the 2^nd^ Department of Medicine Semmelweis University, Budapest, Hungary, the Endocrinology Unit of the Department of Medicine, University of Padua, Italy, the Department of Experimental and Clinical Biomedical Sciences, Endocrinology Unit, University of Florence, Italy and at the Division of Endocrinology, Diabetes, Metabolism and Nutrition, Department of Internal Medicine, Mayo Clinic, Rochester, USA. 6 ACA and 6 ACC samples have been included in the screening cohort, whereas 18 ACA and 16 ACC in the validation cohort. The study was approved by the Ethical Committee of the Hungarian Health Council and informed consent was obtained from all patients involved. All experiments were performed in accordance with relevant guidelines and regulations.

In every operated case, the diagnosis of ACA or ACC was determined by histological examination, whereas the diagnosis of ACA in non-operated cases was based on imaging and follow-up by abdominal computed tomography. The hormonal profile has been investigated in all cases. Tumor stage in ACC was determined based on ENSAT (European Network for the Study of Adrenal Tumors) criteria^43^. The clinical characteristics of both cohorts are summarized in Table 2. Patients did not receive chemo- or radiotherapy before blood collection.

**Table 2:** Characteristics of patients.

Sample number	Tumor type	Cohort	Sex	Age at blood taking (year)	Hormonal activity	Ki-67 (%) or mitotic index (N/10 HPF)	Weiss score	ENSAT Tumor stage
1	ACC	Screening	F	51	Cortisol	25%	9	1
2	ACC	Screening	F	62	Subclinical testosterone	5/10 HPF	7	3
3	ACC	Screening	F	46	Non-secreting	10/10 HPF	6	4
4	ACC	Screening	M	43	Non-secreting	n.d.	6	4
5	ACC	Screening	F	57	Non-secreting	20–25%	5	4
6	ACC	Screening	F	36	Cortisol	n.d.	n.d.	4
7	ACC	Validation	F	67	Cortisol	n.d.	n.d.	4
8	ACC	Validation	F	39	Subclinical cortisol	10%	7	1
9	ACC	Validation	F	57	Non-secreting	n.d.	5	4
10	ACC	Validation	M	80	Non-secreting	n.d.	9	3
11	ACC	Validation	F	56	Testosterone	n.d.	6	2
12	ACC	Validation	M	53	Non-secreting	n.d.	9	3
13	ACC	Validation	F	31	Cortisol	n.d.	6	2
14	ACC	Validation	F	58	Testosterone	2/10HPF	4	3
15	ACC	Validation	F	24	Androgens	10%	5	2
16	ACC	Validation	F	22	Subclinical cortisol and androgens	10%	3	1
17	ACC	Validation	M	62	Cortisol	30%	7	2
18	ACC	Validation	M	48	Cortisol	15%	8	3
19	ACC	Validation	F	38	Cortisol, androgens	70%	8	2
20	ACC	Validation	F	32	Non-secreting	5%	3	1
21	ACC	Validation	F	20	Non-secreting	5	3	4
22	ACC	Validation	M	51	Non-secreting	2%		4
23	ACA	Screening	F	64	Aldosterone	n.d.	n.d.	
24	ACA	Screening	F	73	Non-secreting	n.d.	n.d.	
25	ACA	Screening	F	63	Non-secreting	n.d.	n.d.	
26	ACA	Screening	F	59	Subclinical cortisol	n.d.	n.d.	
27	ACA	Screening	F	77	Non-secreting	n.d.	n.d.	
28	ACA	Screening	F	61	Cortisol	n.d.	n.d.	
29	ACA	Validation	F	38	Non-secreting	n.d.	n.d.	
30	ACA	Validation	F	74	Non-secreting	n.d.	n.d.	
31	ACA	Validation	F	52	Non-secreting	n.d.	n.d.	
32	ACA	Validation	M	29	Non-secreting	n.d.	n.d.	
33	ACA	Validation	M	71	Non-secreting	n.d.	n.d.	
34	ACA	Validation	F	81	Non-secreting	n.d.	n.d.	
35	ACA	Validation	M	63	Non-secreting	n.d.	n.d.	
36	ACA	Validation	M	50	Non-secreting	n.d.	n.d.	
37	ACA	Validation	F	50	Cortisol	n.d.	n.d.	
38	ACA	Validation	F	69	Cortisol	n.d.	n.d.	
39	ACA	Validation	F	46	Aldosterone	n.d.	n.d.	
40	ACA	Validation	M	62	Cortisol	n.d.	n.d.	
41	ACA	Validation	F	33	Cortisol	n.d.	n.d.	
42	ACA	Validation	F	35	Non-secreting	n.d.	n.d.	
43	ACA	Validation	F	65	Non-secreting	n.d.	n.d.	
44	ACA	Validation	F	54	Non-secreting	n.d.	n.d.	
45	ACA	Validation	F	66	Non-secreting	n.d.	n.d.	
46	ACA	Validation	M	68	Non-secreting	n.d.	n.d.	

n.d.: no data, HPF: high power field, F: female, M: male.

### Sample processing and extracellular vesicle isolation

In every case, EDTA-anticoagulated blood was taken from patients and processed for plasma isolation instantly after collection. Plasma was gained by centrifuging whole blood at 1000 x g for 10 minutes at 4 °C. All extracted plasma samples were stored at −80 °C until further use. No hemolysis was observed under these storage conditions.

We used two different approaches for EV isolation. First, EVs were isolated from 200 µl plasma with Total Exosome Isolation (from plasma) Kit (Thermo Fisher Scientific, Waltham, MA, USA) according to the manufacturer’s protocol.

As a second approach, we isolated EVs from blood plasma with differential centrifugation/ultracentrifugation. Cellular components were eliminated with centrifugation (2500xg, room temperature, 15 min), then the platelet-free plasma was diluted to 2x with phosphate-buffered saline (PBS), and was filtered through a 0.8 µm filter (Merck Life Science, Darmstadt, Germany) by hydrostatic pressure to remove remaining platelets and apoptotic bodies. The EVs were pelleted with ultracentrifugation at 100.000xg in an MLA-55 fixed-angle rotor (BC-OptimaTM Max-XP, MLA-55, A/D: 8, Beckman Coulter, Brea, CA, USA) for 1 h at 4 °C from 6 mL of 0.8 µm filtered and 2x PBS-diluted platelet-free plasma samples. The supernatant was discarded and the pellet was resuspended in 90 µl PBS. The solution was treated with Ribonuclease A from bovine pancreas (PN: R6513 by Merck Life Science). The RNase treatment was suspended by Ribonuclease Inhibitor Recombinant (PN: R1158-2.5KU by Merck Life Science). The solution was washed again with 0.1% Bovine serum albumin (BSA) and ProtectRNA RNase Inhibitor 500× Concentrate (PN: R7397 by Merck Life Science) and centrifuged at 100,000xg, 4 °C for 1 h (BC-OptimaTM Max-XP, MLA-55, A/D: 8, by Beckman Coulter). The solution was treated with 700 µl QIAzol Lysis Reagent (ID: 79306, Qiagen GmbH, Hilden, Germany). Samples were stored at −20 °C until further processing.

### Transmission electron microscopy (TEM)

To evaluate EVs isolated by ultracentrifugation, paraformaldehyde (4%) in 0.01 M PBS was added onto the pellets for 60 min at room temperature. Following washing with PBS, we used 1% OsO4 for 30 minutes (Taab, Aldermaston, Berks, UK) for postfixation. The fixed pellets were rinsed with distilled water, then graded ethanol was used for the dehydration of the preparations. We applied 1% uranyl-acetate in 50% ethanol for 30 min for block staining, and the preparations were inlayed in Taab 812 (Taab). Ultrathin sections were made following the overnight polymerization of blocksat 60 °C. We have used Hitachi 7100 electron microscope (Hitachi Ltd., Japan) equipped with a Megaview II (lower resolution, Soft Imaging System, Germany) digital camera for the analysis of the sections.

### Flow cytometry

Direct measurement of (EVs) by flow cytometry is limited because of the small size of blood plasma EVs Therefore we have first adsorbed the EVs onto the surface of formaldehyde/sulfate Latex-Beads (Molecular Probes, Eugene, OR, USA). In brief, EVs were resuspended in PBS and were loaded onto 3.8 μm diameter 4% v/v% beads. Two percent BSA was used as a negative control. Then the samples were incubated overnight at 4 °C under agitation (5 × g). EV-coated beads were incubated for 1 hour with 2% BSA, and then 300 μl 0.9% NaCl solution was added and samples were centrifuged for 10 minutes at 2000xg. The beads were resuspended in 200 μl 100 mM glycerol solution and were incubated for 30 minutes at room temperature. CD9 (FITC, Sigma;#:SAB4700095), CD63 (PE, Sigma;#:SAB4700218), CD81 (FITC, molecular probes;#:A15753) and annexin V (FITC, SONY; #:3804530) were investigated. Altogether we have evaluated 6 samples (3 from ultracentrifugation and 3 isolated by Total Exosome Isolation (from plasma) Kit. Flow cytometry was perfomed by FACSCalibur (BD Biosciences, San Jose, CA, USA). For data analysis we have used FlowJo Software (Tri Star Inc., Ashland, OR, USA).

### Size distribution of extracellular vesicles

The size distribution of extracellular-vesicles were characterized by dynamic light scattering (DLS) on a Zetasizer Nano S instrument (Malvern Instruments Ltd, Malvern, UK) on 4 samples isolated by Total Exosome Isolation (from plasma) Kit (Thermo Fisher Scientific). From the intensity fluctuations of a 633-nm laser light scattered at high angle from the freely moving suspended particles, their diffusion constant was obtained. Size distribution was calculated by using the Stokes-Einstein equation by the built-in algorithms of the instrument’s software. Z-average values are displayed, which represent the primary and most stable parameter produced by DLS technique^44^ and are recommended for quality control reports (ISO 22412:2008). Z-average value represents a good approximation of hydrodynamic diameter of well dispersed particles. Polydispersity index (PDI) is an estimate of the width of the distribution which is calculated from the cumulants analysis.

Light scattering was measured at 25 ± 1 °C. Extracellular-vesicles (refractive index: 1.370) were suspended in 1x sterile phosphate buffer (PBS). The viscosity of PBS at 25 °C was 0.8882 cP and the refractive index was 1.330. The sample was equilibrated at 25 °C for 1 min.

### miRNA expression profiling

Total RNA was instantly extracted from EVs (previously isolated by Total Exosome Isolation (from plasma) Kit) using Total Exosome RNA and Protein Isolation Kit (Thermo Fisher Scientific). In the validation cohort, 5 µl of 5 nM Syn-cel-mir-39 miScript miRNA Mimic (Qiagen GmbH) as a spike-in control for purification efficiency was added before the addition of Acid-Phenol: Chloroform. The final total RNA elute was 50 µl. RNA was stored at -70 °C until further processing.

miRNA expression profiling of EVs was performed altogether on 12 samples (6 ACA and 6 ACC) using high-throughput real-time qPCR. The samples were processed strictly following the Megaplex Pools for microRNA expression Analysis protocol given by the manufacturer as previously described (PN: 4399721B, Thermo Fisher Scietific). In brief, the previously isolated RNA was reverse-transcribed using the TaqMan microRNA Reverse Transcription Kit (PN 4366597, Thermo Fisher Scientific) and Megaplex RT primers Human Pool A v2.1 (PN: 4399966, Thermo Fisher Scietific) on Proflex Base PCR System (Thermo Fisher Scientific). Before the Real-Time PCR reactions, we have run the preamplification reactions of cDNA using TaqMan Preamp Master Mix (2x) (PN: 4384266, Thermo Fisher Scietific) and Megaplex PreAmp Primers (10x), Human Pool A v2.1 (PN: 4399233, Thermo Fisher Scietific) according to the manufacturer’s instructions. The diluted PreAmp product was mixed with TaqMan Universal PCR Master Mix, No AmpErase UNG, 2x (PN: 4324018, Thermo Fisher Scietific) and was loaded on TaqMan Array Human MicroRNA Cards v2.0 pool A (PN:4398977, Thermo Fisher Scientific) and analysed on QuantStudio 7 Flex Real-Time PCR system (Thermo Fisher Scientific). Each sample was assayed with an A card resulting in the analysis of a total of 377 human miRNA.

TLDA raw data analysis, threshold settings and basic statistics were perfomed by Applied Biosystems qPCR Analysis Modules on Thermo Fisher Cloud (https://www.thermofisher.com/hu/en/home/cloud.html). Due to the lack of *cel-miR-39* or other universally accepted reference for circulating miRNA studies on the TaqMan Array Human MicroRNA Cards system^45^, we skipped normalization of raw data applying a certain endogenous control gene on screening results, therefore the normalized quant for the reference sample then reflected the fold change indicated between the different samples directly. We used ACA samples as Reference Biological Group, confidence level was set to 95% and Benjamini-Hochberg false discovery rate correction was applied for p values. The maximum allowed CT value was set to 40 and microRNAs showing no amplification until cycle 40 were considered as not expressed. We used Fisher’s exact test to compare the prevalance of expression of those miRNAs which were detected not in all samples (expressed in more ACC samples compared to ACA and vice versa).

### Validation of individual miRNAs by quantitative Real-Time PCR

Two miRNAs showing a tendency being differently expressed between ACA and ACC samples on TaqMan low density array (TLDA) cards have been subjected to validation by RT-qPCR. Among these, *hsa-miR-101* (002253) and *hsa-miR-483-5p* (002338) were selected for validation by real-time RT-qPCR on altogether 34 samples (18 ACA and 16 ACC patients). Total RNA (5 µl) was reverse transcribed using specific TaqMan microRNA Assays (PN 442795, Thermo Fisher Scientific) and the TaqMan MicroRNA Reverse Transcription Kit (Thermo Fisher Scientific) on Proflex Base PCR System (Thermo Fisher Scientific). Quantitative RT-PCR was performed by TaqMan Fast Universal PCR Master Mix (2x) (PN: 4352042, Thermo Fisher Scientific) on a 7500 Fast Real-Time PCR system (Thermo Fisher Scientific) according to the protocol of manufacturer for TaqMan Small RNA Assays (PN 4364031E, Thermo Fisher Scientific) with minor modifications. Samples were run in triplicate. Negative control reactions did not include cDNA templates. For the evaluation of the data we used the dCT (CT) method (−dCT values = −[CT of target miRNA −CT of internal control miRNA])^46^ using Microsoft Excel 2010 (Microsoft Corporation, Redmond, WA, USA).

### RNA isolation from vesicles isolated by ultracentrifugation and quantitative Real-Time PCR

In the case of EVs isolated by differential centrifugation/ultracentrifugation, RNA was isolated from vesicles (n = 4 ACA, n = 4 ACC) using RNeasy Mini Kit (ID:74104, Qiagen GmbH) and RNeasy MinElute Cleanup Kit (ID:74204, Qiagen) according to the manufacturer’s protocol. The analysis of vesicular RNA was performed by capillary electrophoresis (Agilent Small RNA Kit (CN:5067-1548) on Agilent 2100 Bioanalyzer, Agilent Technologies, Santa Clara, CA, USA). 1 µl RNA in solution was analyzed according to the manufacturer’s protocol. The reverse transcription and qRT-PCR was performed as previously described above. 0.5 µl of 5 nM Syn-cel-miR-39 miScript miRNA Mimic (Qiagen GmbH) was added to the isolated RNA elution during reverse transcription. We have evaluated the expression of *hsa-miR-483-5p* in ACA and ACC patients relative to *cel-miR-39* as reference.

### Statistical analysis

Statistical analysis of high-throughput real-time PCR data and RT-qPCR data was performed by GraphPad Prism 7.02 (GraphPad Software, Inc., La Jolla, CA, USA). For the identification of diffentially expressed miRNAs between ACA and ACC groups, Student’s t-test or Mann-Whitney test were used depending on the results of Shapiro-Wilk normality test. P-values < 0.05 were considered significant.

To identify miRNA markers applicable for diagnosis, receiver operating characteristics (ROC) analysis was performed by GraphPad Prism 7.02.

## Electronic supplementary material

Supplementary Table S1

Dataset 1

Dataset 2

Dataset 3
