# Supplementary material for: Evaluation and diagnostic potential of circulating extracellular vesicle-associated microRNAs in adrenocortical tumors
Source: Sci Rep. 2017 Jul 14;7:5474. doi: 10.1038/s41598-017-05777-0 (PMC5511159; doi:10.1038/s41598-017-05777-0)
Supplement: Supplementary file 1 — Supplementary Table S1 [file 41598_2017_5777_MOESM1_ESM.pdf]

Supplementary Table S1

The percentage of positive beads by flow cytometric analysis

Evaluation and diagnostic potential of circulating extracellular vesicle-associated microRNAs in adrenocortical tumors

Pál Perge, Henriett Butz, Raffaele Pezzani, Irina Bancos, Zoltán Nagy, Krisztina Pálóczi, Gábor Nyírő, Ábel Decmann, Erna Pap, Michaela Luconi, Massimo Mannelli, Edit I. Buzás, Miklós Tóth, Marco Boscaro, Attila Patócs, Peter Igaz*

	Annexin V		CD81		CD9		CD63	
	**FL1-H+ %**	FL1-H- %	**FL1-H+ %**	FL1-H- %	**FL1-H+ %**	FL1-H- %	**FL2-H+ %**	FL2-H- %
beads-BSA AX.V-FITC.	**0,47**	99,50						
beads-BSA CD81-FITC.			**0,52**	99,50				
beads-BSA CD9-FITC.					**0,50**	99,50		
beads-BSA CD63-PE.							**0,50**	99,50
Exosome Isolation Kit sample 1	**2,51**	97,50	**0,75**	99,30	**0,72**	99,30	**0,52**	99,50
Exosome Isolation Kit sample 2	**6,59**	93,40	**0,15**	99,90	**0,64**	99,40	**0,74**	99,30
Exosome Isolation Kit sample 3	**1,20**	98,80	**0,76**	99,20	**0,55**	99,40	**0,87**	99,10
Ultracentrifugation sample 1	**65,80**	34,20	**3,90**	96,10	**34,60**	65,40	**4,31**	95,70
Ultracentrifugation sample 2	**76,40**	23,60	**2,96**	97,00	**37,40**	62,60	**24,60**	75,40
Ultracentrifugation sample 3	**20,80**	79,20	**1,92**	98,10	**11,60**	88,40	**1,48**	98,50
